
JOURNAL INFORMATION
==============================
NLM Title Abbreviation: Insights Imaging
Journal ID: Insights Imaging
NLM Journal ID: 101532453

Insights into Imaging

EISSN: 1869-4101
Publisher: Springer

ARTICLE INFORMATION
==============================
PMCID: PMC7987509
PMID: 33759029
DOI: 10.1186/s13244-021-00975-x
Article ID: 975
Article version: 2
Subjects: Meeting Abstracts

EuSoMII Virtual Annual Meeting 2020 Book of Abstracts

Electronic publication date: 2021 Mar 24
Publication date: 2021 Mar
Volume: 12
Issue: Suppl 1
Issue sponsor: EuSoMII 2020 is the annual meeting of the European Society of Medical Imaging Informatics. Publication costs were not supported by sponsorship.
Electronic Location ID: 39
Copyright: © The Author(s) 2021
Copyright year: 2021
License: Open Access This article is licensed under a Creative Commons Attribution 4.0 International License, which permits use, sharing, adaptation, distribution and reproduction in any medium or format, as long as you give appropriate credit to the original author(s) and the source, provide a link to the Creative Commons licence, and indicate if changes were made. The images or other third party material in this article are included in the article’s Creative Commons licence, unless indicated otherwise in a credit line to the material. If material is not included in the article’s Creative Commons licence and your intended use is not permitted by statutory regulation or exceeds the permitted use, you will need to obtain permission directly from the copyright holder. To view a copy of this licence, visit http://creativecommons.org/licenses/by/4.0/.
License URL: https://creativecommons.org/licenses/by/4.0/

Conference name: EuSoMII Annual Meeting 2020 - How to Prepare for the Digital Age?
Conference Acronym: https://www.eusomii.org/
Conference location: Virtual
Conference date: 24 October 2020
issue-copyright-statement: © European Society of Radiology (ESR) 2021

==============================

EuSoMII

### Insights into Imaging

Education and strategies in European radiology

EuSoMII Virtual Annual Meeting 2020 Book of abstracts / Volume 12 / Supplement 1 / March 2021

EuSoMII Annual Meeting 2020

How to prepare for the digital age?

October 24, 2020

ESR EUROPEAN SOCIETY OF RADIOLOGY

### Annual Meeting 2020

### How to Prepare for the Digital Age?

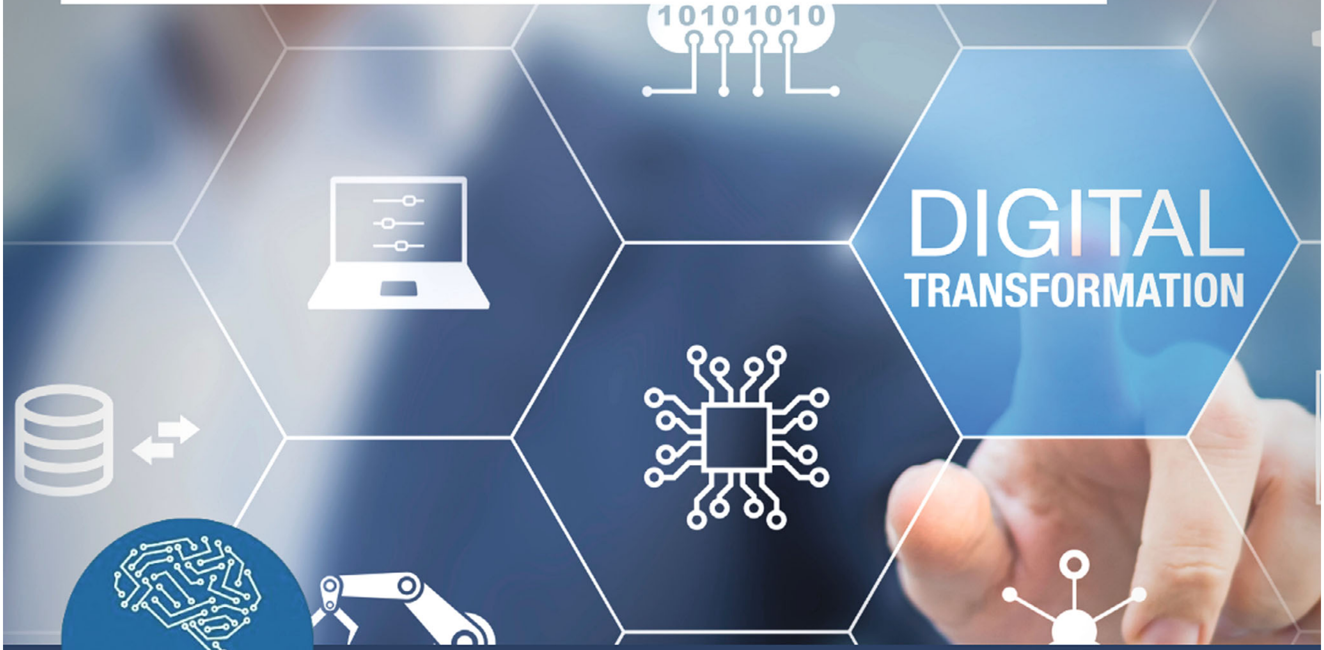

EuSoMII

October 24, 2020

### EuSoMII Annual Meeting 2020
### Book of Abstracts

EuSoMII European Society of Medical Imaging Informatics
Am Gestade 1, 1010, Vienna - info@eusomii.org - www.eusomii.org

### SS 1

### Multi-stage AI analysis supporting prostate cancer diagnosis

*J. Suchanek, A. W. Rix, A. H. Mehan, C. J. L. Doran, A. R. Padhani, C. Kastner, T. Barrett, E. Sala*

Short Summary: A new artificial intelligence (AI)-based system, designed to support clinical interpretation of prostate MRI, shows promising performance compared to radiology studies and CAD/AI literature.

Purpose/Objectives: Investigate whether AI could improve specificity of patient selection for biopsy and biopsy target identification, and assist segmentation, in the prostate cancer diagnostic pathway.

Methods and materials: A multi-stage AI-based system was developed for MRI analysis, and trained with the NCI-ISBI 2013 Challenge, PROMISE12 and PROSTATEx datasets, split into training, validation and held-out test sets.

Clinically significant prostate cancer (csPCa) was defined as Gleason≥3+4 disease. Accuracy metrics were computed on validation and held-out test sets, and compared with literature on prostate MRI studies and CAD/AI models.

Results: To support patient selection for biopsy as a rule-out test, sensitivity identifying patients with csPCa was 93% (95% CI 82-100%), specificity 76% (64-87%), NPV 95% (88-100%), and AUC 0.92 (0.84-0.98), evaluated with biparametric MRI (bpMRI) data from the combined PROSTATEx validation and held-out test sets (prevalence 35%, 80 patients). Performance was higher on the held-out test set (40 patients). Similar AI/CAD publications report 93% sensitivity using held-out/blinded data at specificity between 6%-42%. In major studies, radiologists' sensitivity per-patient was 88-93%, specificity 18-68%, and NPV 76-97%, with Likert/PI-RADS ≥3 defined as positive. Note that methodological and dataset differences and test set size limit comparisons.

For identifying biopsy targets, the AI system detected csPCa lesions with per-lesion sensitivity 94% (85-100%), specificity 71% (61-89%), NPV 97% (93-100%), and AUC 0.89 (0.83-0.95), in the same combined PROSTATEx development validation/test set (128 lesions, 80 patients). Performance was higher on the held-out test set.

For prostate gland segmentation to support analysis, PSA density evaluation, and fusion biopsy, the system showed 92% average Dice score, against radiologist ground-truth segmentations, on held-out test cases from the PROMISE12 dataset (10 patients). This is comparable to the state-of-the-art.

The AI system performed similarly in the above tasks when evaluated using multiparametric MRI (mpMRI) data.

Conclusion: The results suggest the AI system has promising specificity, sensitivity and NPV to help csPCa-free patients avoid biopsy, to support biopsy targeting, and to provide high-quality automated segmentations.

Disclosure: AW Rix and E Sala co-founded Lucida Medical, the company developing this AI system. While regulatory approvals are in progress, the system is not currently available and is described here in retrospective research use.

Keywords: Prostate cancer; MRI; AI; CAD; biopsy; segmentation

### SS 2

### How do medical physicists perceive artificial intelligence?

*Oliver Díaz, Gabriele Guidi, Federica Zanca*

Short Summary: Artificial Intelligence (AI) is rapidly changing medical imaging. Multidisciplinary teams of radiologists, engineers, computer scientists and other related professions are involved in the design and validation of clinical AI tools. But, what about medical physicists? Are they the great forgotten ones? Would they like to participate in AI projects? A flash survey was conducted to answer these and more AI-related questions within the Medical Physics community.

Purpose/Objectives: To assess current perceptions, practises and education needs pertaining to AI in the medical physics field.

Methods and materials: A 25-questions international survey (Google Forms) promoted by the working group on AI of the European Federation of Medical Physics (EFOMP) was organised between February and March 2020. Statistical analysis was performed using the Welch's two sample t-test (SciPy 1.5.0)

Results: A total of 219 medical physicists (average age 42±10 years old; 29% female) from 31 countries took part in the survey. Participants showed an average knowledge about AI (2.3±1.0 on a 1-to-5 scale). About 80% of participants think that AI will improve their profession and 96% of them expressed high interest in improving their AI skills. More than 80% expect AI to be part of their professional curriculum. Despite of this, 64% of them have a limited participation or do not participate in AI projects yet. Significantly fewer female participants are leading AI projects.

Conclusion: Medical physicist perceived AI as a positive resource and are willing to improve their AI skills, thus AI courses specific for medical physicists should be organised. Medical physicists should be also involved in the multidisciplinary teams of AI projects. Special actions should be considered to increase the involvement of female medical physicists.

Disclosure: N/A

Keywords: Artificial Intelligence, International Survey, Medical Physics, Professional Education

Insights Imaging (2021) 12 (Suppl 1): 39
https://doi.org/10.1186/s13244-021-00975-x
Published Online: 24 March 2021

### SS 3

### Hyperlinks in radiology reports: improved efficiency in comparing current with previous oncologic imaging examinations and high user satisfaction.

*A.W. Olthof, H.P. Stallmann.*
*Treant Health Care Group, Department of Radiology, Dr G.H. Amshoffweg 1, Hoogeveen, The Netherlands*

Short Summary: This research evaluates the efficiency and usability of the workflow of finding lesions in previous CT examinations by using hyperlinks in a simulation study, a user enquiry, and an analysis of radiology reports.
Radiologists can use hyperlinks in previous reports to improve efficiency in looking up lesions for response evaluation in oncologic imaging.

Purpose/Objectives: Comparison with previous examinations is a key component of response evaluation in oncologic imaging. For reliable response evaluation, radiologists should compare the current study with the baseline study, and if applicable the nadir study. Finding a particular study, series, and lesion to compare with is prone to errors and time-consuming. The PACS allows the application of hyperlinks in radiology reports. The application of this functionality in daily-practice by radiologists has not been systematically assessed. Our purpose is to develop and evaluate a strategy for accurate and efficient oncologic CT evaluation using hyperlinks in radiology reports.

Methods and materials: This study describes a specific part of the reporting workflow regarding looking up lesions in previous examinations for comparative purposes. We parallel the conventional and hyperlink-enhanced approach by demonstrating a schematic representation and performing a simulation study. We assessed the usage of hyperlinks by a survey among radiologists and checked radiology reports of oncology CTs in the periods 27 June-27 September 2016 and 27 June- 27 September 2020.

Results: The simulation study demonstrates that radiologists can achieve up to a fivefold timesaving for looking up previous lesions by using the hyperlink workflow compared with the conventional workflow to find and compare with previous studies. The response rate of the survey was 86% (12/14). The results indicated that 92% of radiologists are familiar with hyperlinks in radiology reports, 83% are making hyperlinks in their reports, 83% use hyperlinks to identify previous examinations, and 92% use hyperlinks in multidisciplinary team meetings. At a 5 point scale, nine radiologists (75%) answered the question on usefulness with 5 (very useful). The other three radiologists gave as answers 1 (not useful), 3 (neutral) and 4 (useful). The percentages of reports of oncology CT's containing hyperlinks in the three-month periods in 2016 and 2020 were 80% (99) and 86% (85) respectively ($p = 0.013$).

Conclusion: Usage of hyperlinks in PACS integrated reporting contributes to accurate and efficient radiology reporting in oncologic imaging. Radiologist value the functionality of hyperlinks high.

Disclosure: None

Keywords: Hyperlinks; Radiology reporting; PACS; Oncologic Imaging.

### SS 4

### Training radiologist on artificial intelligence: A critical review and opportunities for future

*Mohammad Hosein Rezazade Mehrizi[1], Erik Ranschaert[2], Floor Schuur[3]*

1. *Associate Professor, School of Business and Economics, Knowledge, Information and Innovation, KIN Center for Digital Innovation, Vrije Universiteit Amsterdam*
2. *Radiologist and A.I. Project Leader at Elisabeth-TweeStedenziekenhuis (ETZ), Tilburg, the Netherlands; Chairman of EuSoMII, guest professor at UGent*
3. *Vrije Universiteit Amsterdam*

Short Summary: As artificial intelligence (AI) becomes a significant area for augmenting and supporting radiology work, radiologists need to actively engage in learning about AI and acquiring the skills needed for working with these systems. However, AI is not yet part of the formal radiology curriculum, nor is systematically trained as part of professional training. At the same time, there are many initiatives for offering training opportunities on AI for radiologists.

Purpose/Objectives: We seek to understand: How far are these initiatives in covering the various topics and skills that radiologists need? What are the aspects that the training programs are missing (which we do not see in the current programs)? Who are the active actors in offering the training programs? And how these initiatives can be further expanded for deep, systematic learning about AI?

Methods and materials: In our recent research, we examined 100 training programs offered to radiologists on AI. We analyze the training programs based on their content, delivery mode, offering agents, instructors, target audience, and ways of legitimization.

Results: Our systematic analysis of these training programs shows that the majority of them focus on the basic concepts, offered in a passive mode. Professional institutions and commercial companies are mostly active in offering these programs, while academic institutes play a relatively marginal role. Finally, we see that the content of these programs often focusses on generic aspects of AI and its potential use-cases; yet, practical and contextual aspects of 'how to critically and effectively work with AI' are quite limited.

Conclusion: Further training programs need to focus on how radiologists can learn to work with the AI applications at work, critically evaluate and supervise them, and develop their skills for their future career.

Disclosure: The full paper of this study is already accepted by the European Radiology Journal.

Keywords: Artificial Intelligence (AI), Machine Learning, Radiology Practice, Training Program

### SS 5

### Deep learning natural language processing in radiology reporting: feasible for automated guideline adherence monitoring for communication of critical findings

*A.W. Olthof (1, 2), P.M.A. van Ooijen (2, 3), L.J. Cornelissen (2, 4)*
*(1) Treant Health Care Group, Department of Radiology, Dr G.H. Amshoffweg 1, Hoogeveen, The Netherlands*
*(2) University of Groningen, University Medical Center Groningen, Department of Radiation Oncology, Hanzeplein 1, Groningen, The Netherlands*
*(3) University of Groningen, University Medical Center Groningen, Data Science Center in Health (DASH), Machine Learning Lab, L.J. Zielstraweg 2, Groningen, The Netherlands*
*(4) COSMONiO Imaging BV, L.J. Zielstraweg 2, Groningen, The Netherlands*

Short Summary: A deep learning natural language processing pipeline was trained and tested with a dataset of radiology reports for the evaluation of communication of critical findings. The excellent results demonstrate that the method is feasible for automated guideline adherence monitoring.

Purpose/Objectives: According to guidelines, radiologists have to document the communication of critical findings in the radiology report. Insight in adherence to these guidelines is important because that clarifies whether correct actions follow critical findings without delay. Manual evaluation of guideline adherence is labour-intensive. Deep learning natural language processing can classify radiology reports, but its performance to monitor guideline adherence is unknown. Our purpose is to build a deep learning natural language processing pipeline and assess its performance in evaluating critical findings guideline adherence in radiology reporting.

Methods and materials: Radiology reports with the documentation of communication of critical findings were retrieved from the PACS by a text-mining query and manual selection on the presence of critical findings. The 4375 anonymized reports were manually assessed by a radiologist for the following four items: documentation of to whom, how, and when was communicated, and whether a dedicated structured reporting template was used for this documentation. A binary score was assigned for each item. The dataset was randomized and split in a 90% training set and 10% testing set. A bidirectional encoder representations from transformers (BERT) pipeline was build using the simple transformers library in Python.

We evaluated model performance by assessing sensitivity, specificity, negative predictive value, positive predictive value, area under the curve and f-score.

Results: *Dataset descriptive statistics*

The prevalence of positive cases in the dataset (n=4375) were 16% (683) for the WHO-item, 28% (1221) for the WHEN-item, 38% (1671) for the HOW-item, and 25% (1101) for template usage.

*Model performance*

The model was trained for 7 epochs, and model performance was evaluated on the test set after each epoch. After epoch 5, the highest overall test performance was found, so that version of the model was used to assess the final model performance.

Model performance metrics on the test set ranged from 0.82 – 0.99 for the WHO-item and WHEN item, from 0.79 - 0.93 for the HOW-item and from 0.95 – 0.99 for template usage.

Conclusion: Deep learning NLP model performance is excellent for automated monitoring of guideline adherence to document critical findings.

The BERT-pipeline can evaluate other radiology reporting metrics as well, to contribute to value-based healthcare.

Disclosure: None

Keywords: Deep learning; Natural language processing; Radiology reporting.

### SS 6

### May that app be with you

*Giovanni Rinaldi*

Short Summary: We all have a smartphone with us during the day. Medical apps could help physicians in accessing trusted informations. I have created RadiologyBox, a free to use mobile app where the user will find a lot of resources divided for categories and specialities.

Purpose/Objectives: Everyday we can access a lot of medical informations thanks to internet, but we have to select the most important ones. You can download a lot of apps, but how many of these are useful?

Methods and materials: I have developed my mobile app using the free no code tool named glideapps; the app is easy to use and free for android and iOS.

Results: In building my project, I asked the collaboration of the awesome radiological twitter community, obtaining 27,000 impressions, 4,000 engagements, 351 profile clicks and 47 replies. All these data were put inside the app named Radiology Box where you will find a lot of radiological resources: articles, atlases, softwares and websites divided for categories and specialities.

You as a user can also upload a new resource directly inside the app helping me in update this project.

Conclusion: Medical apps in general and RadiologyBox could help us in learning and working in an easy way. And the advantage is the it is all free.

Disclosure: No relevant disclosure.

Keywords: Mobile apps, educational tools

### SS 7

### Continual Learning in Diagnostic Radiology: Scenarios, Challenges and Perspectives

*Camila González*, *Anirban Mukhopadhyay*

Short Summary: Deep Learning systems deployed for diagnostic radiology should learn continuously. We present an overview of different continual learning strategies and clarify the difference between preventing catastrophic forgetting and achieving positive backwards transfer.

Purpose/Objectives: Evaluating deep learning algorithms in a static setup does not provide a reliable estimate of the performance they would show in the clinical routine. During deployment, datasets are created continuously with variations in acquisition protocol and equipment. For privacy reasons, older datasets may no longer be available after a time, but a system would ideally take advantage of new data. In continual learning, the data base consists of several distinct datasets that arrive sequentially, and the goal is to train a model that performs well on all.

Methods and materials: Continual learning methods usually follow one of the following strategies. Rehearsal methods store a subset of examples from previous tasks and periodically interleave these during training. As storing examples may not be possible in some settings, pseudo-rehearsal methods instead generate artificial examples similar to those seen at earlier stages. Sparse-connectivity approaches discourage overlap between representations by maximizing sparsity, and network growing methods prevent the loss of model capacity by continuously adding new trainable parameters. Finally, regularization approaches calculate an importance value for each parameter and penalize the divergence from them weighted by the importance.

Results: Neural networks trained using stochastic gradient descent on sequentially arriving data adapt too strongly to properties present on the last batches. For data like that seen in the initial stages of training, this causes a significant drop in performance known as catastrophic forgetting. Most continual learning methods focus on preventing this. However, the end goal of continual learning should lie in leveraging ensuing data so that the performance on datasets seen at the start improves with further training. This behavior is often referred to as positive backwards transfer and is unfortunately rarely reported in practice.

Conclusion: The evaluation of deep learning algorithms for medical imaging should more closely emulate the performance in the clinical practice. This includes training models continuously instead of artificially creating similar training and testing datasets. Continual learning methods would ideally not only prevent catastrophic forgetting but also continuously learn robust, domain-independent features that improve the performance of the model on all observed data.

Disclosure: The authors declare that they have no known competing financial interests or personal relationships.

Keywords: Continual learning, catastrophic forgetting, domain shift

### SS 8

### CNN BASED artificial intelligence ALGORITHM COMPRAISE-G FOR VERTEBRAL FRACTURES DETECTION

*Alexey Petraikin*, *Zhanna Belaya, Victor Gombolevsky, Mikhail Belyaev, Kristina Sergunova, Zlata Artyukova, Anastasia Kiseleva, Vladimir Kondratenko, Maxim Pisov, Ekaterina Akhmad, Dmitry Semenov, Nikita Kudryavtsev, Lyudmila Nizovstova, Anton Vladzymyrskyy, Sergey Morozov*

Short Summary: Osteoporosis vertebral fractures (OVFs) are common complications (6-20% population older 50 y.o.) and often unreported on chest and body CT scans. Only 16-32% OVFs have been correct indicated in radiological reports. We developed an artificial intelligence (AI) algorithm (the Comprise-G AI model) which has a high diagnostic capability for automatically detecting the compression fractures of the vertebral bodies and improving osteoporosis management.

Purpose/Objectives: To develop AI convolutional neural networks (CNN) based model for automated diagnosis of compression fractures of the thoracic and lumbar spine based on chest CT images.

Methods and materials: We used anonymized data from 160 patients (> 60 y.o., 127 with VOF), who underwent chest CT. The training group (100 patients) and the test group (60 patients). 2066 vertebral bodies (483 with compression fractures) were labeled by seven readers and then validated by two radiologists 12- and 35-years' experience. We use the Genant semiquantitative method for determining the severity of vertebral body compression. The training data set was used to develop the Comprise-G AI model, which is a combination of two CNN: the first based on is U-net and based on VOLOv3 for the second neural network.

Results: The Comprise-G model demonstrated sensitivity of 90.7% (95% CI [83.4%; 98.0%]) and specificity of 90.7 [83.4%; 98.0%] on the 5-fold cross-validation data of the training dataset; sensitivity was 83.2% [73.7%; 92.7%] and specificity was 90.0% [82.4%; 97.6%] on the test group for patient's level (2-3 Genant compression stage). AUC ROC for the cross-validation and test data were 0.974 and 0.956, respectively. In vertebrae level the model demonstrated sensitivity: 91.5% [90.0%; 93.0%], specificity: 95.2% [94.0%; 96.4%], AUC ROC: 0.981 on the cross-validation data; for the test data sensitivity: 79.3% [76.5%; 82.1%], specificity: 98,7% [97.9%; 99.5%], AUC ROC: 0.978.

Conclusion: The CNN based AI Comprise-G model demonstrated high diagnostic capabilities in detecting the compression fractures of the vertebral bodies. A promising aspect of this approach is the opportunistic screening for osteoporosis based routine abdomen and chest CT.

Disclosure: No conflict of interest

Keywords: Artificial intelligence, Comprise-G, vertebral fractures, computed tomography, convolutional neural networks

### SS 9

### BMRI EXAMINATIONS USING INDUCTIVE MACHINE LEARNING TO CLASSIFY BREAST LESIONS REVISITED

*Evangelia Panourgias[1], Evangelos Karampotsis[2], Georgios Dounias[2]*
[1]First Department Of Radiology, Medical School, NKUA, Greece.
[2]Management and Decision Engineering Laboratory (MDE-Lab), School of Engineering, University of the Aegean.

Short Summary: The purpose of our study is to investigate the contribution of artificial intelligence in medical decision-making processes by developing intelligent and functional medical knowledge extraction systems that may be utilized for accurately diagnosing breast cancer, using clinical and imaging data.

Purpose/Objectives: To identify the effectiveness of one or a combination of breast MRI (bMRI) descriptors in using inductive decision trees to classify breast lesions.

Methods and materials: The dataset included data from 77 patients: clinical (1 variable), imaging (13 variables) derived from breast MRI's, and surrogate markers (6 variables) derived from biopsy examinations. Breast MR exams were acquired on a 1.5T and a 3T scanner. They were reviewed by two independent readers, who were blinded to the histology results and clinical data of each patient.

The selection of the appropriate input variables (for the machine learning algorithm used in this work) was made based on the clinical significance of each variable and with the ultimate goal of optimal performance of the algorithm (evaluation criterion: classification accuracy)

Classification models were developed using the method of inductive decision trees (IDT).

Induction classification trees were converted to IF / THEN classification rules.

The overall approach was evaluated using the "Leave – One – Out" method and utilizing existing medical knowledge.

Results: One classification rule states that 'if the lesion has MASS Morphology and the ADC map displays low signal, then it is a HER-2 positive tumor' with a confidence level of 83.3%. Another rule states 'If the patient is over 50 years old, with a non-mass enhancing lesion (NME) and the presence of a feeding vessel, then the Ki-67 is over 26%'. Yet another, 'If there is Peritumoral Edema, the Kinetic Curve is Type II and the lesion is Unifocal, then the tumor is PR negative'. Lastly, 'if the lesion has a NME morphology, low signal intensity on T2WI, the ADC is low and is Unifocal, then it is ER negative'. The last three classification rules have a confidence level of 80%.

Conclusions: Decision trees are a useful clinical tool that improve classification accuracy of selected datasets. Prospective studies on large datasets are needed to further validate the diagnostic performance of the proposed inductive decision trees.

Disclosure: None.

Keywords: inductive decision trees, machine learning, breast MRI, breast cancer

### SS 10

### A survey to understand how easy it is for radiologists to learn about AI

*Andreas Bucher, Anirban Mukhopadhyay, Daniel Pinto do Santos, Sebastian Jestädt*

Short Summary: In recent years Artificial Intelligence (AI) is becoming increasingly relevant towards digital healthcare. But the programming-heavy materials available within the web has become a burden for the healthcare and practitioners who would most likely benefit from it. While it is anticipated that the radiologists will work more closely together with AI in near future, there is a perceived lack of practical learning material to make them "AI-ready". We emphasize here that we are not expecting radiologists to be expert programmers, in the same way that while radiologists learn about MR physics, they don't get trained as physicists. However, adequate practical knowledge is of primary importance to critically appreciate a technology that is supposed to be in daily use of a radiologist's clinical routine. To get a better overview of the inaccessibility of knowledge problem within European Union as described above, we designed a survey. The short survey (takes about 3 minutes) explores different experiences of radiologists during self-study of AI. The first two questions are about what the most difficult part of learning about AI is and when the topic was last addressed. In this way, the experience can be put into a chronological order and knowledge can be gained about what makes it difficult for radiologists to learn. In a further question the participants are then asked more precisely what exactly was so difficult. The possible answers are as follows: Coding intensive, too much gimmick, information overload or nothing specific about radiology for a more detailed analysis. This should help us to identify in which of the four categories the biggest problems are. At the end we will go back to what was perceived as bad in the last time with the applied strategy, so that we can better evaluate failures and work out a solution that avoids these failures.

Purpose/Objectives: The goal of this survey is to understand how easy it is for entry-level enthusiastic radiologists to learn about AI.

Methods and materials: A survey: https://forms.gle/6QzVbn9FdGPrUyucA

Results: Eight radiologists have participated in the survey. We won't describe the detailed results here to ensure a bias-free understanding of the European picture.

Conclusions: Our plan is to develop an e-learning solution for radiologists and other medical professionals that focuses on self-study of AI. This should provide enthusiastic entry-level professionals an opportunity to critically appreciate AI beyond their medical school curriculum.

Disclosure: August 2020

Keywords: AI-Ready, AI, Artificial Intelligence , digital healthcare, learning AI, radiology, self-study

### SS 11

### Development of severity and mortality prediction models for COVID-19 patients at emergency department including the chest X-ray.

*Pilar Calvillo-Batllés, Leonor Cerdá Alberich, Carles Fonfría-Esparcia, Ainhoa Carreres-Ortega, Carlos Francisco Muñoz-Núñez, Laura Trilles-Olaso, Carlos Baeza-Delgado, José Sánchez-García, Ana Gil-Brusola, Rodrigo Blanco-Salado, Alberto García-Ortega, Marta Montero-Alonso, Pilar Estellés-Lerga, Javier Millán-Soria, Cristina Aguado-Codina, Luis Martí-Bonmatí.*

Short Summary: The COVID-19 pandemic is posing a large challenge for health systems, forcing a balance to be found between resource management and safe decision-making. We developed and internally validated severity (AUC-ROC=0.94) and in-hospital mortality (AUC-ROC=0.97) prediction models that could be useful to triage symptomatic COVID-19 patients. Some of the strongest predictors include oxygen saturation, age, and extent score of lung involvement on chest X-ray. These models should be further validated at different emergency departments.

Purpose/Objectives: To develop prognosis prediction models for COVID-19 patients attending an emergency department based on chest X-ray, demographics, clinical and laboratory parameters.

Methods and materials: All symptomatic confirmed COVID-19 patients admitted to our hospital emergency department between February 24th and April 24th 2020 were recruited. Chest X-ray features, clinical and laboratory variables and chest X-ray abnormality indices extracted by a convolutional neural network (CNN) diagnostic tool were considered potential predictors on this first visit. The most serious individual outcome defined the severity level: home discharge or hospitalization ≤ 3 days, hospital stay >3 days and intensive care requirement or death. Severity and in-hospital mortality multivariable prediction models were developed and internally validated. The Youden index was used for model selection.

Results: A total of 440 patients were enrolled (median 64 years; 55.9% male); 13.6% patients were discharged, 64% hospitalized, 6.6% required intensive care and 15.7% died. The severity prediction model included $SatO_2/FiO_2$, age, C-reactive protein (CRP), lymphocyte count, extent score of lung involvement on chest X-ray, lactic dehydrogenase (LDH), D-dimer level and platelets count, with AUC-ROC=0.94 and AUC-PRC (precision-recall curve)=0.88. The mortality prediction model included age, $SatO_2/FiO_2$, CRP, LDH, chest X-ray extent score, lymphocyte count and D-dimer level, with an AUC-ROC=0.97 and AUC-PRC=0.78. The addition of chest X-ray CNN-based indices improved the predictive metrics for mortality (AUC-ROC=0.97, AUC-PRC=0.83).

Conclusions: The developed and internally validated severity and mortality prediction models should be used as triage tools for COVID-19 patients at emergency department.

Disclosure: The authors declare no competing interests.
The authors have not received any funding. Jose Sánchez-García is an employee of Quantitative Imaging Biomarkers in Medicine (QUIBIM SL) whose software has been used in one of the predictive models.
Keywords: COVID-19, chest X-ray, prognosis, predictive models, artificial intelligence.

### SS 12

### Imaging COVID-19 AI initiative

*Laurens Topff, Erik Ranschaert*

*Laurens Topff, MD*
*Department of Radiology, Netherlands Cancer Institute, Amsterdam, The Netherlands*
*l.topff@nki.nl*
*https://orcid.org/0000-0003-1186-3007*

*Erik R. Ranschaert, MD, PhD*
*Department of Radiology, ETZ Hospital, Tilburg, The Netherlands*
*University of Ghent, Ghent, Belgium*
*e.ranschaert@etz.nl*

Short Summary: Imaging COVID-19 AI initiative is a large collaborative effort to develop a deep learning solution for assisted diagnosis of COVID-19 on CT scans, and for assessing disease severity by quantification of lung involvement. The project is initiated by the European Society of Medical Imaging Informatics (EuSoMII). Twenty-six hospitals and two industry partners across Europe have collaborated to collect data and develop a deep learning model. A large and varied dataset of more than 3000 Chest CT-scans was obtained by the project group, containing a mix of COVID-19 positive scans, normal scans and other types of pulmonary infection. Radiologists have been actively involved in the selection and annotation of imaging data. The presentation explains the approach and current state of this multicenter European project.

Disclosure: None.
Keywords: Artificial intelligence, Deep learning, COVID-19

### SS 13

### Low-Powered Deep Learning Models for Radiology Imaging

*Amara Tariq, Aishwarya Harpale, Priyanshu Sinha, Judy W Gichoya, Saptarshi Purkayastha*

Short Summary: Deep learning based models like ResNet, DenseNet, InceptionNet, etc. have revolutionized radiologic image processing. Large memory and computational requirements make deployment of these models on low-powered Acorn RISC Machine (ARM) devices challenging. We worked on developing low-powered and compressed versions of deep models focused on chest X-rays.

Purpose/Objectives: Deep learning models for radiology image processing based on architectures like convolutional neural networks (CNN), 3D-CNN, U-Net, ResNet, InceptionNet, MobileNet, etc., have been able to achieve high performance for tasks such as tumor classification, detection of hemorrhage, detection of disease such as pneumonia, edema, cardiomegaly, and more recently COVID-19. Real-world deployment of such models to low-powered devices such as ARM devices, is still a challenge as these models require large amounts of computational power and memory for training and evaluation. Our work is focused on developing low-powered versions of high performing disease detection models, especially focused on chest X-ray datasets, opening up the scope for implementation of such models on devices like mobile-phones and embedded boards with comparable accuracy.

Methods and materials: Explored compression techniques include **i) Quantization:** model parameters conversion to low-precision data-types like **i-a) int8** or **i-b) float16** by rounding off scaled parameters. Run-time selection of scaling factor implies **i-c) dynamic** quantization. **ii) Pruning** removes redundant model links with minimal performance reduction. **iii) Hybrid** technique combines pruning and quantization.

We used **i) RSNA pneumonia detection dataset** (30000 X-rays, binary classification) with **i-a) DenseNet201** and **i-b) InceptionNet-V3**, and **ii) Chest X-ray14 dataset** (112120 X-rays, 14 findings, multi-class classification) with **ii-a) ChexNet**.

Results: For RSNA dataset, InceptionNet holds its performance steady under almost all compression techniques. Dynamic quantization provides the most size reduction (251MB to 21 MB) with 0.1% performance improvement. For Chest X-ray14 dataset, CheXNet behaves similarly under Float16 and dynamic quantization. Dynamic quantization achieves the most reduction in size (29.4MB to 7.3MB) while keeping performance stable. DenseNet for RSNA dataset tends to suffer relatively worse performance reduction for various compression techniques. The best reduction in size is achieved by int8 quantization (211MB to 18MB) with about 7% reduction in performance (84.3% to 76.9%). Float16 quantization results in minimum performance reduction (0.1%).

Conclusions: Our experimentation shows that by intelligent application of compression techniques, model computational requirements can be reduced without significant sacrifice of performance.

Disclosure: Authors have no conflicts of interest to disclose, financial or otherwise.

Keywords: Low-powered deep learning models, Low-powered radiology image annotation

### SS 14

### Semi-automatic Labelling of Radiology Images

*Amara Tariq, Kislay Singh, Priyanshu Sinha, Saptarshi Purkayastha, Judy W Gichoya*

Short Summary: Datasets for training AI algorithms for radiology images are expensive to collect and annotate requiring thousands of hours. Moreover, model performance may be different in real life, when new data characteristics are encountered. Various techniques have been developed to curate large datasets for medical AI, including use of weak labels, classifying radiology reports using natural language processing and reader studies to annotate the radiology images. There is an opportunity for utilizing human in the loop techniques for annotating datasets, whereby the radiologist modifies a model generating annotation for faster and more accurate labeling. To support such workflow, we developed an easy-to-use and intuitive visualization and labelling tool called SALORI (Semi-Automatic Labelling Of Radiology Images).

Purpose/Objectives: The purpose of this work is to develop a semi-supervised annotation tool for radiology images where a radiologist can be kept in the loop during the annotation process for fine-tuning automatically generated annotations. Such a tool has the potential for generating large labelled data sets of radiology images that can be further used for training AI applications in radiology.

Methods and materials: Our tool employs deep learning based inference models to process chest X-rays for tasks like detection and segmentation, and then visualizes their output such as bounding boxes for detection and segmentation masks. It also allows radiologists to modify these bounding boxes and segmentation masks.

Results: Our efforts resulted in the addition of the following features in SALORI

- Web-interface for visualization of deep models' outputs
- Support for bounding box and segmentation mask
- Human Annotation/modification of visualized bounding boxes and segmentation mask
- Export of annotated result in csv format
- Ability for integration of any deep learning model

Conclusions: Our tool opens the scope for large annotated dataset collection to facilitate AI research, in addition to providing intuitive visualization for current AI algorithms as assistance for radiologists.

Disclosure: Authors have no conflicts of interest to disclose, financial or otherwise.

Keywords: Semi-supervised annotation, radiology image annotation, human-in-the-loop radiology image annotation

### SS 15

### Legalock Badge: strong certification applied to the radiology report, on your smartphone

*Carmelo G. Puglisi MD, UOC Radiodiagnostica Arnas Garibaldi Catania*
*Placido Romeo MD, UOC Radiodiagnostica, Taormina Hospital - ASP Messina, Italy*

Short Summary: Legally proved identity certification to validate documents like radiologic reports or medical certifications is an issue nowadays because of costs of third party intermediation and flexibility of digital signs solutions. We describe a certification service that acquires media and text through edge devices (smartphones, IoT) and encapsulates them into a managed ledger database where every transaction is cryptographically encrypted and immutable.

Purpose/Objectives: Purpose of this paper is to evaluate the feasibility and utility of Legalock service in the daily use of medical and radiological workflow.

Methods and materials: Legalock is an apple mobile Ios based app providing a real time identity certification. It's based on blockchain technology an end to end platform where the source at the edge is enforced by biometrics, other sensors and real time features powered by the internet that ensures instance subscriber identity, time and place of the activity and locks this to the attached files .

Blockchain guarantees sign authenticity via a cryptographic sequence of hashes (blocks) and timestamps that realize a sequence that can't be duplicated or hacked. This key is locked to data (images, radiologic reports, certificates) and cryptographically linked information are stored and secured together. Nowadays this technology has been used to allow cryptocurrency transactions, contracts exchange or to share financial and public records. We are exploring use cases in radiology.

Results: Legalock has been released as a beta app working under IOS operative System for apple mobile devices and allows you to produce a certification of a document linked to anything (a dicom file, an image or other) with a tracked file of the legal proof that the subscriber and time and location are real. This technology was recognized as the winner of the italian startup competition Telecom TIM WCap 2016-17 award.

Conclusions: We are confident that this promising technology will change medical and radiological workflow making easier every step and allowing sensible data sharing between health professionals and patients

Disclosure: Dr. Puglisi Carmelo G. is the inventor of Legalock service
Dr. Placido Romeo nothing to disclose

### SS 16

### Three-dimensional printing in endovascular surgery: preoperative planning and simulation in a complex case of pericallosal aneurysm

Short Summary: Aneurysms of pericallosal artery are rare conditions, which in some cases are difficult to treat surgically due to anatomical variations or distal location. Three-dimensional printing (3DP) is a new technological tool that allows to realize a physical model from medical imaging data, as generated for example by computed tomography. We report the case of a 68-year-old female with pericallosal aneurysm successfully treated with endovascular embolization, after the development of a 3DP vascular model. In our case, the 3DP model played a crucial role as regards the planning and simulation of the surgical procedure, thus facilitating the treatment of the aneurysm.

(Cite this article as: Puglisi CG, Galvano G, Biondi A, Basile F, Quartararo S, Borzì AM,M.Vacante, A.Scavone. Three-dimensional printing in endovascular surgery: preoperative planning and simulation in a complex case of pericallosal aneurysm. J Radiol Rev 2020;7:000–000. DOI: 10.23736/S2723-9284.20.00044-8)

### SS 17

### Preliminary results of an international Survey on Virtual meetings

*D'Anna G.[1], Chen M.M.[2], Tali E.T.[3], Patel A.[4], Kotsenas A.L.[5], Van Goethem J.[6,7], Garg T.[8], Hirsch J.A.[9], Martì-Bonmatì L.[10], Gaillard F.[11],12, Ranschaert E.[13]*

*[1]Radiology Unit, Humanitas Mater Domini – Castellanza (VA), Italy.*
*[2]Department of Diagnostic Radiology, The University of Texas MD Anderson Cancer Center, Houston, United States.*
*[3]Department of Radiology, Gazi University School of Medicine, Ankara, Turkey.*
*[4]Department of Radiology, Liberty Hospital, University of Missouri-Kansas City School of Medicine, St. Louis, Missouri, United States.*
*[5]Department of Radiology, Mayo Clinic, Rochester, Minnesota, United States.*
*[6]Department of Biomedical Sciences, Antwerp University Hospital, Antwerp, Belgium.*
*[7]Department of Radiology, AZ Nikolaas, Sint-Niklaas, Belgium.*
*[8]Seth GS Medical College & KEM Hospital, Mumbai, India.*
*[9]Massachusetts General Hospital and Harvard Medical School, Boston, MA, United States.*
*[10]Medical Imaging Department, La Fe University and Polytechnic Hospital, Valencia, Spain.*
*[11]Faculty of Medicine, Dentistry, and Health Sciences at the University of Melbourne, Parkville, Australia.*
*[12]Department of Radiology, the Royal Melbourne Hospital, Parkville, VIC, Australia.*
*[13]Department of Radiology, Elisabeth-Tweesteden Hospital (ETZ), Tilburg, The Netherlands.*

Short Summary: Between June and August 2020 we performed a 16 questions online survey about preferences on virtual meetings. Preliminary data showed that people appreciated the opportunity to attend online the radiological meetings, with some meeting design changes.

Purpose/Objectives: We explored perceptions and preferences about the conversion of in-person meetings to virtual ones. Here we show some preliminary data.

Methods and materials: We prepared a survey (in English) that was available online between June and August 2020. The survey comprised of 16 questions about preferences regarding online conference attendance.

It and was targeted to radiologists, residents and medical students.

Results: We received a total of 508 answers from 71 countries.

The largest number of answers were submitted from Italy and India.

Most people at the time of the survey had already attended a virtual meeting (80%) and would like to attend further virtual meetings in the future (97%).

The ideal duration of a virtual meeting was 2-3 days (42%) or half a day (32%).

The preferred time format was a 2-4 hours meeting, with 1-2 breaks (43%).

Conclusions: Respondents appreciated the opportunity to attend online the most important radiological meetings; however, they generally would prefer a change in meeting design.

A deeper analysis of subgroup data, for example by country or age, would be very useful.

Keywords: Virtual Meetings, survey
